# Draft Genome Sequences of Salmonella enterica subsp. enterica Serovar Dublin Strains from St. Nectaire and Morbier Cheeses Characterized by Multilocus Variable-Number Tandem-Repeat Analysis Profiles Associated with Two Fatal Outbreaks in France

**DOI:** 10.1128/MRA.01361-18

**Published:** 2019-01-03

**Authors:** Sabrina Cadel-Six, Marie-Leone Vignaud, Manal Mohammed

**Affiliations:** a Université Paris-Est, Marne-la-Vallée, France; b ANSES, Laboratory for Food Safety, *Salmonella* and *Listeria* Unit, Maisons-Alfort, France; c School of Life Sciences, University of Westminster, London, United Kingdom; University of Maryland School of Medicine

## Abstract

We report here the draft genome sequences of 2 Salmonella enterica subsp. enterica serovar Dublin strains from St. Nectaire and Morbier cheeses having multilocus variable-number tandem-repeat analysis (MLVA) profiles identified during the fatal outbreaks that occurred in France in 2012 and 2015 to 2016, respectively. These draft genome sequences will help uncover the virulence determinants in invasive S. Dublin strains.

## Abstract (precis)

We report here the draft genome sequences of 2 Salmonella enterica subsp. enterica serovar Dublin strains from St. Nectaire and Morbier cheeses having multilocus variable-number tandem-repeat analysis (MLVA) profiles identified during the fatal outbreaks that occurred in France in 2012 and 2015 to 2016, respectively.

## ANNOUNCEMENT

Salmonella enterica subsp. enterica serovar Dublin belongs to the nontyphoidal Salmonella (NTS) group, which causes primarily self-limiting gastrointestinal illness in humans; however, it has been associated with a higher occurrence of bacteremia and systemic illness (1, 2). S. Dublin is adapted to cattle (3), and it can be transmitted to humans through the consumption of contaminated unpasteurized milk or dairy products, including cheese made with raw milk (4). Human outbreaks of S. Dublin have been reported in many European countries and the United States (5–8). The outbreaks of S. Dublin reported in France in 2012 and in 2015 to 2016 due to St. Nectaire and Morbier cheese consumption, respectively, resulted in serious illness and a high mortality rate (5, 9).

Here, we announce the draft genome sequences of 2 S. Dublin strains (2015LSAL00258 and 2014SAL02972) isolated from a St. Nectaire producer and Morbier cheese manufactory, respectively. The multilocus variable-number tandem-repeat analysis (MLVA) profile 14-8-10-7-5-3 of S. Dublin isolate 2015LSAL00258 from St. Nectaire cheese is related to the human cases of the 2012 outbreak in France (5). On the other hand, the MLVA profile 15-8-10-7-5-3 of S. Dublin isolate 2014LSAL02972 from Morbier cheese is associated with the fatal outbreak in 2015 to 2016 (9).

Bacterial strains were grown overnight on nutrient agar at 37°C, and then genomic DNA was extracted using the QIAamp DNA minikit (Qiagen, UK). Genomic DNA quality and quantity were checked using gel electrophoresis and the Qubit quantification platform (Invitrogen, USA), respectively. Genomic DNA libraries were prepared using a Nextera XT library preparation kit (Illumina, San Diego, USA), following the manufacturer’s protocol. Whole-genome sequencing (WGS) of multiplexed libraries was carried out on the Illumina HiSeq platform using a 250-bp paired-end (PE) protocol.

Illumina sequencing yielded a total of 1,447,855 PE reads for the St. Nectaire isolate (2015LSAL00258) and 898,743 for the Morbier cheese isolate (2014LSAL02972). The FastQC toolkit (http://www.bioinformatics.babraham.ac.uk/projects/fastqc/) was used to evaluate the quality of the sequencing data. Adapter sequences were removed using the ea-utils package (https://expressionanalysis.github.io/ea-utils/). *De novo* assembly was generated from all the Illumina sequence data using SPAdes version 3.11 (10).

The size of the draft genome of the St. Nectaire cheese isolate (2015LSAL00258) is 4,878,335 bp and consisted of 26 contigs, with an overall GC content of 52.1% and *N*_50_ value of 494,440 bp. The size of the draft genome of the Morbier cheese isolate (2014LSAL02972) is 4,878,331 bp, and it consisted of 27 contigs, with an overall GC content of 52.1% and an *N*_50_ value of 494,440 bp. The draft genome for each isolate was annotated using Prokka version 1.12 (11).

The genetic basis of virulence of S. Dublin is not well characterized, and it is not understood how bacteria can evade the human immune system and cause invasive disease. These draft genome sequences will help uncover the virulence determinants in S. Dublin. A detailed report of these genomic features will be addressed in a future publication.

### Data availability.

The raw sequence reads of the two S. Dublin isolates from St. Nectaire cheese (2015LSAL00258) and Morbier cheese (2014LSAL02972) have been deposited in the European Nucleotide Archive (ENA) under accession numbers ERS2767808 and ERS2767809, respectively. The draft genome sequences of isolates 2015LSAL00258 and 2014LSAL02972 are available under accession numbers ERR2818363 and ERR2818364, respectively.
