# Erratum for Cadel-Six et al., “Draft Genome Sequences of Salmonella enterica subsp. *enterica* Serovar Dublin Strains from Raw Milk Cheeses Characterized by Multilocus Variable-Number Tandem-Repeat Analysis Profiles Associated with Two Outbreaks in France”

**DOI:** 10.1128/MRA.01590-19

**Published:** 2020-02-13

**Authors:** Sabrina Cadel-Six, Marie-Leone Vignaud, Manal Mohammed

**Affiliations:** a Université Paris-Est, Marne-la-Vallée, France; b ANSES, Laboratory for Food Safety, *Salmonella* and *Listeria* Unit, Maisons-Alfort, France; c School of Life Sciences, University of Westminster, London, United Kingdom

## ERRATUM

Volume 8, no. 1, e01361-18, 2019, https://doi.org/10.1128/MRA.01361-18. Page 1: The article title should read as given above.

Page 1, abstract, line 2: “St. Nectaire and Morbier cheeses” should read “two raw milk cheeses.”

Page 1, abstract, lines 3 and 4: “fatal outbreaks” should read “outbreaks.”

Page 1, lines 8 and 9: “St. Nectaire and Morbier cheese consumption, respectively, resulted in serious illness and a high mortality rate (5, 9)” should read “consumption of two raw milk cheeses resulted in serious illness among humans (5, 9). Although deaths were reported, there was no confirmation that cause of death was attributable to *S.* Dublin infection.”

Page 1, lines 11 and 12: “from a St. Nectaire producer and Morbier cheese manufactory” should read “from a raw cheese producer and a cheese manufactory.”

Page 1, lines 13 and 14: “isolate 2015LSAL00258 from St. Nectaire cheese is related” should read “isolate 2015LSAL00258 is related.”

Page 1, lines 15 and 16: “isolate 2014LSAL02972 from Morbier cheese is associated with the fatal outbreak” should read “isolate 2014LSAL02972 is associated with the outbreak.”

Page 1, lines 24 to 26: “reads for the St. Nectaire isolate (2015LSAL00258) and 898,743 for the Morbier cheese isolate (2014LSAL02972)” should read “reads for the isolate 2015LSAL00258 and 898,743 for the isolate 2014LSAL02972.”

Page 2, line 4: “the draft genome of the St. Nectaire cheese isolate (2015LSAL00258)” should read “the draft genome of the isolate 2015LSAL00258.”

Page 2, lines 6 and 7: “the draft genome of the Morbier cheese isolate (2014LSAL02972)” should read “the draft genome of the isolate 2014LSAL02972.”

Page 2, lines 15 and 16: “isolates from St. Nectaire cheese (2015LSAL00258) and Morbier cheese (2014LSAL02972)” should read “isolates 2015LSAL00258 and 2014LSAL02972.”

